# Perceptions of preschool teachers of the characteristics of gifted learners in Abu Dhabi: A qualitative study

**DOI:** 10.3389/fpsyg.2022.1051697

**Published:** 2022-11-01

**Authors:** Ahmed Mohamed, Hala Elhoweris

**Affiliations:** College of Education, United Arab Emirates University, Abu Dhabi, United Arab Emirates

**Keywords:** gifted, preschool, teacher, perceptions, Abu Dhabi, characteristics

## Abstract

Considerable evidence supports that preschool education is a milestone stage for children. Nonetheless, systematic preschool gifted education programs rarely exist in public elementary schools. The current study explored the perceptions of 16 preschool teachers (general and special education teachers) from seven public schools in Abu Dhabi, United Arab Emirates (UAE) regarding their views about various components of gifted education for preschool children. Qualitative analyses, using the inductive data analysis method, revealed several themes such as (a) the concept and identification of giftedness, (b) characteristics of gifted preschoolers, (c) preschoolers’ problem-solving skills, (d) the communication and social skills of gifted preschoolers, resources/services offered by the school to serve gifted preschoolers, (e) enrichment programs available for gifted preschoolers, (f) inclusive education for gifted preschoolers, (g) twice-exceptional preschoolers, and (h) governmental support. The results of this study may help advocate for infusing more services and programs related to the identification and education of gifted preschoolers in public schools. The findings identified the need to have an abundance of assessment tools and enrichment programs that can empower preschool teachers to cater for giftedness.

## Introduction

Teachers play a major role in the development of gifted preschoolers, not just academically but holistically. They must be well-informed, trained, and supported so that they are motivated and equipped to carry out quality educational interventions. It is also salient that they hold positive perceptions and attitudes toward giftedness and gifted education as these were found to affect the way they teach children (Kettler et al., 2017b). Thus, the views and feedback of teachers must be considered to ensure that their teaching needs are addressed and that their best teaching practices are recognized and documented. Teachers need to be given the chance to take part in evaluating gifted education programs in their respective schools and the country.

There is no universally accepted definition of giftedness. Several waves of the conception of giftedness existed (Sternberg and Kaufman, 2018). For example, general intellectual ability represented in intelligence assessments was used to identify gifted children. Domain-specific models (e.g., Thurstone) identified more specific mental abilities involved in intellectual performance with general ability at the very top. The systems model involved conceptions of giftedness that could entail other psychological variables such as creativity (e.g., Renzulli’s three-ring definition). Developmental models (e.g., Gagné’s model) focused on the talent development process through the interaction of environmental influences, non-intellective variables, and learning.

Recent studies that looked into teachers’ perceptions of gifted students and gifted education programs revealed that educators’ understanding of giftedness is primarily based on their educational background, training, and philosophy (Kettler et al., 2017b), and their experiences working closely with regular and gifted children. For instance, Margrain and Farquhar (2012) determined that their teacher respondents were not able to define giftedness in a single or cohesive manner, as their beliefs and understanding of it varied.

Nordström (2022) explored the conditions of identifying gifted preschoolers in the Swedish school system by interviewing 10 preschool teachers and 5 principals. The participants were asked about their conceptions of giftedness and the obstacles to meeting gifted preschoolers’ needs. The results showed participants’ lack of knowledge about giftedness and the ‘gifted’ labeling issues in society. Antoun (2022) examined Lebanese primary school teachers’ perceptions of the education of gifted students. The findings showed that cultural context had an impact on teachers’ choices and perceptions in relation to gifted education. Antoun et al. (2020) explored Lebanese primary teachers’ perceptions of gifted students. The findings showed that teachers had positive attitudes toward gifted education but limited awareness of the evidence-based Western practices of gifted education. Antoun et al. (2022) examined Lebanese teachers’ perceptions in relation to education approaches used to identify and serve gifted students in primary schools. The results showed that teachers had a lack of awareness of international practices. El Khoury and Al-Hroub (2018) examined Lebanese primary teachers’ perceptions in relation to the gifted students’ characteristics. The researchers proposed multidimensional identification model that combines psychometric and dynamic assessment, based on the Lebanese teachers’ perceptions toward giftedness and the characteristics of gifted learners (Al-Hroub and El-Khoury, 2018).

Other studies showed that teachers often associate giftedness with more positive characteristics (Moon and Brighton, 2008), and defined it based on gifted students’ exemplary skills and abilities, such as cognitive capacities, motor skills, and social skills (Yazici et al., 2017). Overall, teachers often explained that the skills that these children possess are significantly more developed compared to children’s chronological age.

Teachers more commonly describe gifted preschool children in terms of their advanced general cognitive abilities – that they are generally intelligent (Konrad and Gabrijelcic, 2015). In some studies, nonetheless, teachers noted specific intellectual abilities that they found significantly advanced among gifted children. These include strong reasoning skills, a broad fund of knowledge, a wide vocabulary (Moon and Brighton, 2008), reading skills and reading comprehension, problem-solving, attention and memory, speed in processing, and creativity (Ogurlu and Çetinkaya, 2012; Dal Forno et al., 2015; Yazici et al., 2017). Teachers believe that these competencies were developed experientially in stimulating home environments. For instance, parents expose their children to books, bring them on trips, or help them learn at home (Moon and Brighton, 2008).

Some gifted children were also noted to possess social skills that are more developed than other children. More specifically, they were described as sociable, able to relate well with peers, can make friends relatively easily (Grant, 2013; Yazici et al., 2017), and have a strong connection with their community (Moon and Brighton, 2008). As early as preschool age, they demonstrate empathy, can sympathize with others (Yazici et al., 2017), and exude leadership qualities (Kettler et al., 2017a; Yazici et al., 2017).

Teachers likewise noted that some of their students were reportedly advanced in some areas of physical development. Some gifted children’s physical or psychomotor skills developed faster than most children their age (Yazici et al., 2017). For instance, parents in Ogurlu and Çetinkaya (2012) study, reported that their gifted children walked earlier or toilet-trained faster. Some also noticed their children’s heightened sensitivity to stimuli, such as light, sound, or smell.

Kettler et al. (2017a) identified several challenges for preschools in setting up and running gifted education programs. For one, a number of preschools have no clear policies on gifted education, which follows that the school’s administration, teachers, and staff are not completely aware of the guidelines for best practices in gifted education. Aside from this, schools find it challenging to find and retain qualified staff, citing that it is a challenge to find a competent early childhood teacher with experience and training in gifted education. Meanwhile, untrained staff or teachers were found unwilling to provide gifted services (Kettler et al., 2017b).

Limited resources such as budget, space, and time pose a challenge in providing gifted education services at the preschool level (Kettler et al., 2017a). Schools that do not consider gifted education as a priority with a limited budget for funding activities and procuring materials. School operators and teachers also noted the lack of space or classrooms specifically dedicated to gifted education programs. The most limited resource that teachers cited was time. Creating policies, deciding on practices, and training on gifted education require time and effort from teachers who are already loaded with work. Likewise, finding or developing curriculum and materials for gifted preschoolers was seen as time-consuming and effortful.

One notable consideration that some preschools have about establishing gifted programs at the early childhood level is the tendency of children’s development to change in a couple or more years (Kettler et al., 2017a). Teachers claim that whereas children may appear advanced for their age at the preschool level, their performance may just be at par when they reach the primary level. Moreover, teachers have reservations about accepting children younger than preschool age, specifically those below the typical school age of 5 years old, even if they exhibit advanced academic abilities. This is due to the belief that younger children benefit best from building relationships with their same-age peers, and that children below 5 years old may not have the emotional maturity to cope with the demands of school (Margrain and Farquhar, 2012).

Despite this generally unseemly feedback regarding the status of gifted education at the early childhood levels, preschool teachers deem that working with gifted preschoolers is important and necessary (Cosar et al., 2015; Konrad and Gabrijelcic, 2015). Aside from honing talent, establishing gifted education services in preschool may help young children and their families deal with challenging behaviors that come with giftedness. If giftedness is detected in preschool, difficult behaviors, such as emotional sensitivity, restlessness, or boredom, may be addressed early (Kettler et al., 2017b).

In addition, teachers uphold that gifted education should be included and treated as special education, as gifted children have needs that are different from their age mates and are not addressed by mainstream education (Margrain and Farquhar, 2012). Hence, this implies that governments’ respective education sectors or Ministries of Education may reconsider the scope of special education to include gifted education.

Professional development training on gifted education was found to contribute to teachers’ attitudes and perceptions toward this program. More specifically, teachers who are more accepting of establishing gifted education programs in their institutions are those who have training in special needs education. In a study conducted by Vreys et al. (2018), teachers felt more confident with their skills in providing educational interventions for the gifted, and in applying differentiation techniques after they underwent extensive training on gifted education.

Teachers have varied views of the processes of identification, assessment, and the conduct of in-school services for gifted children. Teachers recognize the value of proper identification of gifted students as early as preschool years (Yazici et al., 2017). Whereas giftedness is typically identified at the primary levels, teachers claim that it is possible to identify the potentially gifted even at an early age (Margrain and Farquhar, 2012; Konrad and Gabrijelcic, 2015). This process is regarded as important by most teachers and they acknowledge the significance of their role as they are the first and constant companions of children in school. Teachers are likely to be the first to spot proficiency and talent in their students as they work with them in class (Senicar and Senicar, 2018).

In a study done by Senicar and Senicar (2018), teachers expressed that they feel confident in their ability to recognize a gifted child by mere observation and evaluation of their class work. There are teachers, however, who feel that they are ill-equipped to identify gifted children. They believe that the process of identification requires greater skills training or having an additional staff or teacher to assist them with this (Konrad and Gabrijelcic, 2015). In Margrain and Farquhar (2012) study, some teachers are not keen on doing a formal assessment for identification but are open to observing children in the classroom.

The learning environment of gifted preschoolers should be enriching in itself, and one filled with a variety of activities, and materials that children can work with (Grant, 2013). However, the school community’s conflicting beliefs about gifted education may pose a challenge to its implementation (Kettler et al., 2017a). Some educators believe that labeling and segregating gifted children is unfair to children, and placing them in higher grade levels bring more disadvantage to their development.

Whereas the method of differentiation has been evaluated as one of the more effective interventions for gifted children, some school administrators and teachers have reservations about mixing regular classroom curriculum and modified curriculum. They noted concerns about allotting equal attention to both curricula and serving the needs of all students in the classroom (Kettler et al., 2017a). Moreover, some schools that adhere to a developmentally appropriate or age-appropriate curriculum perceived gifted services as digressing from this pedagogy, hence, may not be completely aligned with programs for the gifted.

Conversely, Grant (2013) noted that more than creating effective in-school services for gifted preschoolers, teachers should prioritize coming up with programs that make every child feel emotionally secure in school and help them build healthy social relationships. It was found that children felt most emotionally secure and intellectually stimulated when these children are genuinely engaged by their teachers in conversations about topics that they find interesting and stimulating. Moreover, teachers believe that helping children socialize and play with their peers is more important than skill development and that it may be beneficial to not highlight they are being exceptional to avoid deliberately differentiating regular and gifted learners (Margrain and Farquhar, 2012).

With the availability of information about gifted individuals and gifted programs, and through the advocacies of organizations for the gifted, people’s understanding of giftedness and their appreciation for gifted services have evolved. Schools began adopting educational programs and interventions to cater to the special needs of gifted learners. Likewise, the past decade has seen a growth in studies on giftedness and gifted education, mostly at the primary and secondary grade levels. There is, however, a paucity of studies on gifted education programs at the early childhood level (Kettler et al., 2017a). Most of the studies on giftedness in preschool revolve around the rigors of identifying potentially gifted children, their characteristics, and early childhood educational institutions’ current gifted programs and services.

Gifted education in the UAE is still developing. The UAE has established several distinctive endeavors to nurture giftedness. Examples include the Hamdan bin Rashid Al Maktoum Foundation for Distinguished Academic Performance, the Emirates Association for the Gifted, and the Abu Dhabi Department of Education and Knowledge (ADEK). Also, the UAE government declared 2015 as the year of innovation. The country celebrates innovation in March of every year. Although ADEK is infusing some promising professional development programs and several enrichment programs, these efforts are almost dedicated to elementary, middle, and high school students. Identification and enrichment opportunities for gifted preschoolers are limited. The COVID-19 pandemic also imposed several challenges concerning helping preschool teachers to identify and nurture giftedness in preschoolers.

It can be noted that there is a dearth in the literature examining in-school gifted services at the preschool level. Other than educational interventions, such as differentiation and curriculum compacting, there was no other specific literature on mainstream school practices that allow for the discovery and further development of potentially gifted preschoolers’ skills and talents. Similarly, literature on the identification of gifted students focused on traditional methods of testing and observations in the classroom. Other non-traditional methods of identification that teachers employ in and out of the classroom have not been widely studied.

Considering that teachers are the everyday companions of preschool children in school and the primary implementors of a school curriculum, it is important to heed their perceptions of giftedness and gifted education programs and to obtain their feedback with regard to their perceived competence in implementing gifted services, and experience of support from the school and larger community.

The purpose of this study was to explore preschool teachers’ perceptions of gifted preschoolers, definitions of giftedness, characteristics of gifted students, and services and programs offered to gifted preschoolers. The following questions guided the study:

1. How do you, as a special education teacher, define the concept of giftedness?
2. What are the intellect and non-intellect characteristics of gifted preschoolers?
3. What are the resources or services offered by your school or community to help identify and nurture gifted preschoolers?
4. What are effective grouping options that should be available to gifted preschoolers?
5. Do you have any twice-exceptional (gifted students with ADHD, gifted students with autism, and gifted students with learning disabilities) students in your classroom?
6. Do you think that gifted preschoolers in the UAE are well served? Do you have any suggestions about how to improve identification and enrichment resources for gifted preschoolers?

### Materials and methods

#### Participants

Because this study aimed at determining preschool teachers’ perceptions about the identification and education of gifted preschoolers, a qualitative method was adopted. The qualitative method recognizes that reality is a social construct of giftedness in which the complexity and context of the emerging data must be considered; the participant, not the method, should be the primary focus (Al-Hroub, 2021, 2014). The process of qualitative research is inductive and allows inquiries about a topic by collecting data. The researchers used qualitative content analysis. The nine questions raised in the study were used as the main categories. The deductive approach was adopted for this analysis (Zhang and Wildemuth, 2009). In this study, a total of 16 preschool teachers participated; 12 female teachers were citizens, and 4 teachers were residents/expats. A total of 13 teachers were homeroom teachers and 3 were special education teachers working in preschools. Their teaching experience ranged from 3 to 14 years. The teachers were selected from 7 public schools in Al Ain City, UAE, a city on the Western side of the UAE. The selected teachers worked in public schools funded primarily by the government.

#### Procedure

A total of nine semi-ended interview questions were used in this study. The researchers of this study asked identical questions that were worded so that the answers provided by participants would be open-ended (Turner, 2010). The purpose of the interview was to collect information about teachers’ perceptions of the education of gifted preschoolers (Kvale, 2008). The interview questions were presented in accordance with the purpose of the study so that the participants’ responses would provide insight into the teachers’ perceptions and experiences related to gifted preschoolers. The researchers attended to any useful information that might lead to follow-up questions to further clarify the perceptions (Rubin and Rubin, 2005). Prior to conducting the research study, the researchers obtained the Social Sciences Research Committee’s approval to start recruiting the study participants. A consent form, outlining the purpose of the study and all relevant details, was shared with teachers from the different schools and 16 teachers consented to participate in the focus group discussions. The participants’ information was anonymized and the focus group discussions were conducted by the researchers of this study who did not know the participants.

Due to the current pandemic conditions, the interviews were conducted online using Microsoft Teams. Interviews were conducted using open-ended questions. The researchers collected data from the interviews about teachers’ perceptions of the identification, characteristics, services, and inclusion of gifted preschoolers (Hatcher et al., 2012). Because the interviews were conducted using MS Teams, the interview sessions were recorded for later analysis (Yin, 2011). Then, the recordings were shared with a research assistant who translated the interview transcripts into English. The researchers checked the English version of the transcripts and shared the transcripts with the participating teachers for further member check and accuracy. A few editing remarks have been applied to some parts of the interview. To protect the privacy of the participants, pseudonyms were used instead of real names, and no identifying information related to the participants or schools were mentioned.

The inductive data analysis was used in this study because it rests on getting information based on participants’ experiences (Yin, 2011). The use of inductive data analysis is advantageous as it allows the researcher to generate meaning from the interview data and helps identify patterns and relationships between participants’ responses (Dudovskiy, 2016). The researcher used NVivo 11 to organize and code the data obtained from the interviews. The data were coded and reassembled and themes were generated. The researchers looked for patterns in the responses given by the teachers and recorded the similarities and differences in responses given by the teachers during the interview (Creswell, 2017). Thus, the researchers started to make interpretations and reach conclusions. Open-ended interviews may restrict the generalization of the results as the participants’ responses might not be authentic (Rubin and Rubin, 2005).

## Results

### Special education teachers’ definition of giftedness

Special education teachers defined giftedness as abilities and talents that are advanced or significantly better developed than the normal population. These abilities were described as natural or innate to children, yet may still improve when nurtured. For the teachers, age serves as a benchmark for comparing the gifted with average children. As Teacher 2 concisely put it, “*students possess the capabilities that are considerably above the norm of their age*.” A number of teachers particularly referred to exceptional intellectual capacities, such as the ability to think quickly or to remember information. They likewise recognize that whereas giftedness is usually associated with exemplary cognitive abilities, exceptionality may also manifest in other domains: “*In my opinion, giftedness is obvious in domains like artistic, intellectual, leadership, creative or in some particular academic fields like science, mathematics or arts.”*

### Identifying gifted preschoolers

The teacher respondents noted that identifying gifted preschoolers is a process. It involves a series of steps with several people working together to determine who among the students is high achieving. According to Teacher 4, *“It takes considerable time, patience, and essential knowledge to recognize the students with giftedness. Giftedness identification is not a short and immediate process. From parents to teachers, everybody plays a vital role in determining students with giftedness.”*

Identification utilizes multiple assessment methods. Teachers recognize the importance of their role in this process. When students are in class, teachers screen for gifted ones by carefully observing students at work and evaluating if a child’s performance is exceptional. They also use tests to assess class performance and to identify which level should these students be placed in.

In some classes, parents are also involved in the screening process. Children are not just observed by teachers in the classroom, but parents are likewise asked to observe their children at home. Teachers who took part in the study enumerated signs of giftedness that parents should look for in their children. These include the speed of learning, relative maturity in thoughts and actions, broad vocabulary, advanced reading skills, interest in problem-solving tasks, capacity to express oneself adequately, and preference for the company of older children over same-age or younger peers.

### Students observed gifts

Teachers recognized that giftedness may be manifested in different domains. The respondents’ students demonstrated exemplary cognitive skills, such as memory, processing speed, verbal expression, reading comprehension, and in math reasoning. Excellent memory was the common denominator among their gifted students. The memory that the teachers described in their anecdotes referred to short-term memory, such as identifying and remembering letters, and to long-term memories, such as remembering situations. Teacher 7 described his student, “*One child was able to remember any story that was read to him in the classroom, he was able to retell the same story again without any mistakes, which showed the teacher his excellent memorization abilities.”*

Other gifts that the teachers noted include their students’ ability to work on tasks more quickly than other students, to fluently express themselves orally and in writing, and to solve math problems with relative ease.

### Teachers’ perceived characteristics of gifted preschoolers

Gifted preschoolers were described in terms of their cognitive strengths, their approach to learning, and with reference to their personal qualities. Most gifted preschoolers were described as significantly intelligent. A number of teachers associated giftedness with having an excellent memory and strong verbal abilities. According to the teachers, despite their students’ young age, gifted preschoolers can learn quickly, remember a great deal of information, and can express themselves fluently with their wide vocabulary. Some were identified to have strong math skills and artistic and musical skills.

According to the teacher respondents, gifted preschoolers approach learning differently than their same-age classmates. They are noticeably more inquisitive. They have varied interests and are constantly asking questions. They likewise enjoy tasks that are challenging and require problem solving, like working with puzzles. These behaviors suggest that gifted preschoolers have an innate desire to learn. As Teacher 9 put it, “*They have a great motivation to learn.”* Aside from these, gifted preschoolers were observed to be imaginative and creative. The more they know, the wider their imagination and the more creative they get. Some gifted children are also endowed with positive qualities, such as leadership and a sense of fairness, high self-esteem, strong motivation, high ambition, capacity for emotion regulation, and a good sense of humor.

### Teachers’ views about gifted preschoolers’ problem-solving skills

Teachers believe that, along with critical thinking, the skill of problem solving is a strength that must be cultivated in gifted preschoolers. Teacher 10 emphasized this point by saying, *“Whatever the method of teaching problem-solving and critical thinking skills, the message is the same - to support the development of gifted children’s strengths, I must give them the opportunity to engage them with problem-solving and employ critical thinking.”*

Teachers noted that gifted preschoolers’ problem-solving skills are advanced for their age and grade level. Teacher 12 shared, *“[They] are sometimes more intelligent than their teachers in solving problems, for example, gifted students can provide problem-solving methods for the teachers”.* This was seconded by another teacher, who said that gifted children are even able to answer questions that adults raise. Aside from these, gifted preschoolers were observed to find solutions by trying out different ways, *“If you put a problem for them, they will put several solutions.”* Teacher 10 described children’s problem-solving skills as *“scientific”* perhaps pertaining to being systematic.

One notable finding refers to problem solving as an ability that may be affected by other skills, such as social skills and self-regulation, and traits like self-confidence. Teacher 7 concisely put it, *“Many gifted individuals with high intelligence may fail in practical life if they do not have the emotional intelligence that makes them more able to deal with feelings of failure in frustration, anger, and excitement, and more able to empathize with others, and to use social skills that make them more efficient in solving the problems.”* This response suggested that problem-solving is not limited to cognitive or academic tasks. It also involves working through conflicts in interpersonal relationships.

### Teachers’ assessment of gifted preschoolers’ communication and social skills

Gifted preschoolers differ in their communication and social skills. Teacher 2 explained, *“Some of them like to communicate and has good social skills, some of them do not, and it depends on their personality and characteristics*.” Most of the teachers who participated in the study noted their students’ capacity to communicate and relate well with others if they want to or when the situation calls for it.

Nonetheless, teachers have observed how these children’s giftedness may affect their ability to connect meaningfully with others. More specifically, they are advanced, hence being different in terms of skills and talents, their tendency to have an inflated sense of self-esteem, and their hypersensitivity, which may keep them from making and keeping friends. Because they are intellectually different, *“their talent may act as an obstacle to his social compatibility and prevent good relationships and friendships from being held with others, and he is being ignored and ostracized by his peers.”* In the same vein, some children are disliked because of their tendency to unknowingly elevate themselves from others, as “*they are smarter*”.

Some gifted children tend to be sensitive and emotional. This characteristic may both be helpful and unfavorable at the same time. One teacher noted how some gifted preschoolers’ tendency to get easily hurt when given negative comments, may keep the child from communicating with his/her classmates or teachers. Hence, teachers underscore the importance of developing communication and social skills so that they may grow well-adjusted. In Teacher 5 own words, “*social and communication skills are essential for the preschooler to adjust to society. When a child is brought up with good communication and social skills, the child is able to establish a healthy relationship with others around him.”*

### Resources and services offered by the school and community in identifying gifted preschoolers

Identification of gifted students is a process. Teachers noted several steps that begin with identification and proceed to the placement of students in the classroom. The first step is usually screening, which involves selecting the potentially gifted and talented from among the students. General education teachers are usually the ones tasked to do this. Once screened, identified children are assessed using different methods. The third step is placing identified students in the gifted programs, whether through inclusion or separate special classes.

The assessment of students is multi-method. The use of classroom assessments and specialized tests are among the most common tools employed by schools. Classroom assessments include the teachers’ objective evaluation of the student’s performance in class activities. The tests that some teachers use are the ones provided by their respective schools, the Ministry of Education, or other institutions such as the British Council in Abu Dhabi.

Identified children are also administered standardized tests, such as specialized tests of giftedness. Teacher 9 noted, “*In our kindergarten, we use a Gifted Identification Kit developed by a team of researchers from the USA, which is about ten different learning centers/intelligence, including mathematical, analytical, spatial, and linguistic, whether the child is gifted or not until the gifted child is discovered through them*”. Other norm-referenced tests used by schools to identify gifted preschoolers include intelligence or IQ tests, achievement tests, and early developmental assessments. It can be noted that most of the tests that schools use are those that only assess intellectual abilities. There was no mention of using tests that look into children’s social or emotional development.

Teachers recognize the value of classroom observation in the process of identification. This is most true for teachers whose institutions do not have specific guidelines for screening for giftedness. Teacher 13 shared, *“there is no specific mechanism in kindergarten because we have a system of learning centers, for example, there is a mathematics center, a construction center, a reading center, and a writing center, the child when he/she starts working in the center, the teacher begins discovering the gifted, whether the child has a potential or not.”* Similarly, observations done by teachers in the classroom provide additional information that supplements the results of standardized tests. Teacher 15 described how his/her student’s behavior in the classroom differed from the child’s test results, *“while doing the IQ test most of the time, the IQ test shows different results than expected. As an example, we conducted an IQ test for a child, and the result was that his IQ level was very low, but his cognitive abilities in the classroom were very good according to what the results showed, so we focused on the classroom observation.*”

Some schools employ the use of questionnaires and checklists to assess the students’ behaviors outside the classroom. These tools were completed either by teachers, parents or both. Aside from assessments, gifted children are also identified through school competitions. Contests and competitions serve as means to spot talented students. Aside from services specific to identification, the teachers mentioned other resources that help them serve gifted students. For one, schools, where the respondents teach, have specialized classrooms designed for gifted students. This is where students take specialized classes apart from those that they take with regular students.

The teachers themselves and their work were considered essential resources in serving gifted learners. From identification, and placement, to nurturing the students’ talents, the role of the teachers is very important. As how Teacher 8 put it, “*it is the diligence of the teacher that she communicates with the parents to try to cultivate the gifts that we discovered.”* In addition, teachers consider other professionals working with these students as valuable in assisting gifted learners to adjust well to school and to achieve their maximum potential.

There are teachers, however, who perceive that their schools lack specific and clear guidelines and resources for gifted students and that the screening and academic modifications were done by teachers on their prerogative. When asked about services and resources, teacher 10 answered, *“Nothing! This is the teacher’s job. The school doesn’t do anything. We used to go to a math class so I could challenge him to push further, but there is a center, great. We’re trying to provide extra activities in math.”*

### Enrichment programs for gifted preschoolers in the school or the community

Different schools offer a variety of programs that aim to support and cultivate gifted preschoolers’ talents. To ensure that students receive continuous services, some schools hold in-school enrichment programs throughout the school year. During school days, students attend activities or special classes apart from their regular classes. Gifted learners benefit from differentiated instruction and curriculum modifications, *“If the teachers discover a gifted child in a specific subject such as science or math, then he/she will give different activities/resources to the gifted child according to his abilities and what suits his interests”.* On weekends, they hold scientific workshops of various interests to help foster children’s skills and abilities. There are also courses, such as training and creative pursuits, held during the holidays and summer to ensure year-round guidance for gifted students.

Aside from these, school-based Learning Support Programs intended for all students were found helpful for gifted learners as well. Teacher 4 described learning support activities as “*an integrated program that we use in our school for a gifted and normal student, it is an in-school program. Our dedicated learning support teacher provides one-on-one and small group work activities with the children using a wide range of materials and activities. Such activities focus on art, creativity, math, and language.”*

The respondents noted how valuable school clubs are in developing their skills. *“The most famous programs among the students are the clubs, in the clubs, they have the opportunity to improve their talent under the teachers’ observation,”* said Teacher 15. Clubs may also serve as a venue for children, who share the same passion and interests, to interact and practice their communication and social skills. Likewise, holding fairs, such as Science and Book fairs, function as opportunities for the students to showcase their skills and learn more about their interests. Meanwhile, competitions held in schools were found helpful not just in identifying gifted students but in challenging themselves and nurturing their talents. One teacher pointed out, *“We can choose talented students and we can get them involved in competitions between schools”*. Teachers appreciate the grants/funds that their students receive from external sponsors. “*The Emirates give grants to gifted students”*. These grants may include financial aid for engagement in activities that may further children’s talents or may be given simply as a reward for their exemplary performance.

At the core of these enrichment programs are the teachers who go the extra mile to help their students develop their gifts. When teachers discover talent, they take it upon themselves to nurture their students’ abilities. Teachers do this to the best of their capacity and given the resources that they have in school. Teacher 13 recounted, “*we give children activities according to their levels, but if we discover a gifted student, we give them more activities, for example, we have research and discovery corner, and also a giftedness corner in which thinking skills are greater and more difficult.”*

### Teachers’ perceptions about inclusive classrooms and separate classrooms for gifted preschoolers

Teachers were found to have varying beliefs when it comes to the placement of gifted preschoolers. They justified their answers to this question by stating the advantages and disadvantages of putting gifted learners in an inclusive classroom or specialized classes separate from regular students. Teachers who are in favor of inclusion stated that bringing regular and gifted students together in one classroom may be mutually beneficial for both groups of learners. *“I think regular classes are better because we will have students with different abilities. As a result, other students will encourage typical students to pursue academic excellence. This will lead to academic progress*.” This view was shared by several respondents. Likewise, teachers deemed that this set-up may positively impact gifted students in the sense that they may develop their communication and social skills through interaction with other students. There were, however, identified disadvantages to inclusion. A couple of respondents shared their concerns about how regular classroom instruction and arrangement may curb the development of gifts, especially if gifted students do not receive extra support. Another challenge lies in the teacher’s ability to cater to both regular and gifted students at the same time. Teacher 9 noted, *“Sometimes there’s not enough time to complete in-depth projects that gifted students like to do*.”

Recognizing these challenges, some respondents share the perception that inclusion with adjunct special sessions or classes might be the better learning set-up for gifted preschoolers. Teacher 2 put it, *“gifted students can show and improve their extraordinary talents or activities in regular classroom lessons. But somewhere in mind, I think that they also need a particular class for enhancing their unique talents. They should be trained individually to improve their gifts and talents”.* These teachers recognize the importance of having gifted and regular students together to help the former develop not just academically, but socially and emotionally as well.

### Presence of twice-exceptional students in the classroom

Three teachers who participated in this study currently have at least one student in their class who display exceptional abilities, yet is diagnosed with autism spectrum disorder (ASD). Two teachers said that their gifted students were diagnosed with Attention Deficit/Hyperactivity Disorder (ADHD). Teacher 5 noted that she had a twice-exceptional student in class, however, was not able to specify what was the condition. The rest of the teachers do not have twice exceptional students at the time the study was conducted. Teacher 2 mentioned, “*last year I had a child with an autism spectrum and had speech problems, but he was distinguished in mathematics*”. Teacher 9 concluded, “*I have a student with autism who has little speech capabilities and cannot explain what he needs*.”

### Teachers’ perceptions of the adequacy of services received by gifted preschoolers in the United Arab Emirates

Most of the respondents perceived that gifted preschoolers in the UAE are well-served. They were most satisfied with the way the Emirates and the Ministry of Education have been supporting and advocating for gifted and talented students. The support that they receive from the government is regarded as essential in servicing gifted learners. As teacher 11 described it, “*the students with giftedness are the wealth of our country. Our government has instructed every school to provide specialized training to gifted students. As our government thinks that gifted students can be the strongest pillars of our development, we should make the best students who can make us proud*.” Teacher 7 underscored how UAE has been at the forefront of servicing individuals with special needs, including gifted and talented students, *“UAE is one of the countries that value individuals with disabilities, and they also provide what a gifted student may need in the academic field. UAE has provided the best programs that can be found in the world. Individuals with special needs also get financial support from the government to help them manage their needs, even though they don’t have to pay for the services they get.*

Overall, the teachers believe that the services are adequate and that the needs of students are well addressed – from material and financial needs to quality supervision from teachers and other allied professionals. Teacher 1 furthered that they take pride in UAE having institutions, such as the Hamdan Bin Rashid Al Maktoum Center for Talent and Creativity support the school and the students, “*The ministry of the education serves the discovery of talents (masterpieces) in various fields for example (music, drama, visual arts, poems, and traditional arts). The Emirates give these students, or they can sometimes travel abroad to get more knowledge and experience. We have a lot of these services*.”

There are, however, teachers who believe that some Emirates still lack efforts in addressing micro issues related to supporting gifted learners. For instance, Teacher 15 noted how parents should be helped in understanding and handling the behaviors of gifted children, *“in some ways, I think they should provide our country… I know a lot of parents make their children feel that they do something wrong when they express themselves, and that makes them feel little discouragement for his/her inability”.* Whereas this concern is not directly related to cultivating gifts and talents, teachers recognize how parents’ appreciation of their children’s gifts and their understanding of their children’s tendencies make it easier for teachers to collaborate with parents.

## Discussion

The purpose of this qualitative study was to explore preschool teachers’ perceptions and awareness of the definition, assessment, identification, characteristics, and services provided to gifted preschoolers. A related purpose was to examine preschool teachers’ conceptualization of gifted preschoolers’ problem-solving skills, resources available to serve gifted preschoolers, and programming options. There is an unmet need in both preschool education and gifted education as to which gifted preschoolers are identified and serviced. The researchers followed the constant comparison analysis in data reduction and nine themes portrayed how preschool teachers envision various factors related to the education of gifted preschoolers.

There is no doubt that preschool education is an essential foundation. Research showed that individual differences in intellectual development occur in preschool (Koshy and Robinson, 2006). Also, providing gifted students with appropriate educational programming such as acceleration will yield considerable effects for those who exhibit advanced abilities and cognitive development (McClarty, 2015). The first theme found in the responses referred to the special education teachers’ definition of giftedness. Teachers in this study referred to giftedness as exceptional intellectual abilities manifested in the ability to think quickly or to remember information. This corroborates what Al-Hroub and El-Khoury (2018) concluded, on a study of the perceptions of 150 Lebanese elementary teachers toward gifted students, that giftedness is a combination of three components: high academic performance, high intellectual ability, and social intelligence.

For gifted preschoolers’ characteristics, teachers in this study reported several characteristics such as good memory, processing speed, verbal expression, reading comprehension, and reasoning skills. Most teachers focus on the role of intellectual capacities that make the preschooler distinguished from other students. They also reported some other characteristics such as curiosity and a wide vocabulary. Other personality characteristics include leadership, a sense of fairness, high self-esteem, high motivation, and a sense of humor. It is important that teachers understand the psychological characteristics of identifying giftedness (Porter, 2005). Havigerová and Haviger (2014) posited teachers that such characteristics as pace, autonomy, and attention constitute the gifted child’s personality. Moreover, the characteristics described by teachers resemble those by Renzulli’s Three-Ring Model in which he posited that gifted students possess several characteristics such as having various interests, curiosity, agility in learning, inquiry skills, and above-average performance (Renzulli, 2002). In addition, teachers’ views about the role of school performance in the identification of giftedness are congruent with Gagne’s view about giftedness as a natural ability that is related to the student’s performance in schoolwork (Gagné, 2010).

Teachers stated that gifted preschoolers possess problem-solving skills that are affected by other factors such as self-regulation, social skills, interpersonal relationships, and self-confidence. Also, teachers reported that gifted preschoolers have good communication skills. This corroborates Kıldan (2011) who found that preschool teachers’ definition of giftedness focused on superiority and creativity. Teachers in this study stressed the important role of general education teachers in the identification process which is based on several stages and layers. They pointed out that classroom assessments and teachers’ evaluations of students’ performance in schools are important indicators of students’ giftedness. Teachers also recognized the necessity of having standardized tests, achievement tests, and developmental assessments in the identification process. However, teachers did not focus on the assessment of social and emotional development. They focused on the important role of classroom observation in identifying gifted students. They consider observation techniques in preschool as valuable tools that consolidate other standardized assessments. Teachers also viewed that school clubs, competitions, and activities play an important role in recognizing different gifts.

Nonetheless, teachers reported that there is a lack of clear and specific guidelines and resources for gifted preschoolers and that screening and curricular modifications are conducted through teachers’ endeavors. There is a lack of theoretical clarity in gifted education which leads to fragmented and inconsistent services (Renzulli, 2012) and inconsistent definitions of giftedness (Subotnik et al., 2011). Hence, the absence of gifted education policies and services can be related to the lack of theoretical clarity in gifted education (Kettler et al., 2017b). Teachers reported that learning support programs are helpful for gifted preschoolers. The role of school clubs is highly emphasized by teachers in developing gifted preschoolers’ skills. Competitions held in the schools are also good sources of identifying students with gifts. External funding also helps teachers run programs that cater for gifted preschoolers.

Teachers’ beliefs related to placement options for gifted preschoolers varied in this study. A group of teachers reported that inclusive classrooms can be beneficial for both gifted and regular students. However, a few teachers were concerned that inclusive classrooms may curb the development of gifted students in case they do not receive enrichment opportunities. Also, the teachers raised the role of teacher preparation in catering for both regular and gifted students at the same time. Kettler et al. (2017b) found that teachers perceived a lack of time, space, and money required for running gifted programs. Teachers’ concerns might indicate that “separatist models of gifted education rather than talent development models advocating integrated differentiation” (p. 127). There is a need to provide gifted students with responsive learning environments in preschool settings (NAGC, 2006).

Although teachers in this study reported that they have children with autism spectrum disorder is their classrooms, however, they might not be familiar with how to identify and nurture children with dual exceptionality. Al-Hroub and Whitebread (2008) concluded that many teachers were not accurate about identification of twice-exceptional children.

Teachers in this study also reported that although the available services are satisfactory there is an unmet need to address micro issues related to supporting gifted learners such as parent support. More services should be directed toward the identification and education of gifted students in preschool education. Preschool-gifted education is a neglected field (Barbour and Shaklee, 1998). Kettler et al. (2017b) reported that the lack of information about gifted education services is one of the major barriers. Outdated perceptions of gifted education can be a barrier perceived by practitioners who might be ambivalent about the current recommended practices (NAGC, 2006).

Teachers also focused on the important role of sustainable professional development in enhancing their abilities to identify and nurture gifted preschoolers. Pianta et al. (2016) found that teachers with at least a bachelor’s degree had higher-quality classrooms and more instructional support. Konrad and Gabrijelcic (2015) found that preschool teachers in Slovenia need more opportunities for professional development as they have low self-competence in relation to identifying the personal characteristics of gifted children. Considering their experiences in working with gifted children, teachers are among the primary persons to know how best to help gifted and talented young learners. Whereas most of them were satisfied with the services that UAE provides for gifted individuals, they deem that these can still be continuously enhanced.

Professional development of teachers was among the most recommended step to furthering gifted programs. One teacher emphasized, *“We always recommend to the government to provide classes for the teacher to get to know more about the special education field and the gifted students.”* Teachers need to be trained in all areas of the program – from identification to the evaluation of performance and abilities. Aside from training and other professional development activities, teachers believe that collaborating with colleagues helps them gain insight and learn strategies for teaching gifted students. Hence, teachers should be given more opportunities to link up and converse with fellow teachers through organized activities, such as conventions or fora. A teacher said, *“Teachers should have the opportunity to go and meet other teachers who got a gifted child in their classroom to discuss things and give them some kind of support and how to challenge these children*.”

Aside from peer collaborations, teachers suggested providing schools with more opportunities to work in partnership with the government or with other private institutions in coming up with activities to support the gifted and the talented. More specifically, these institutions may initiate national exhibitions, where students can showcase their talents, or hold workshops and awareness programs for the community, the school, and the families.

Teachers shared a good number of suggestions to help schools boost their gifted programs. At the school level, support may come in the form of acquiring resources, such as assessment tools, and improving the efficiency of identification and placement of gifted students. Teachers also see the value of constantly reviewing the programs to be more responsive to the gifted students’ changing needs. The schools may also consider providing more workshops on giftedness for families and communities and organizing more in-school enrichment programs.

### Implications

The results of this qualitative study warrant some important implications. Most of the teachers’ responses indicate that gifted education services in preschools are still developing and that there should be clear guidelines to identification and service delivery. Teachers need more professional development opportunities in the field of identification and enrichment of gifted preschoolers. During these challenging times as imposed by the COVID-19 pandemic, teachers struggled with providing all students with appropriate schooling because of the lockdown. Accordingly, online learning can play a significant role in providing teachers with key tools that enable them to identify and nurture giftedness in preschoolers. Examples of online professional development opportunities might include webinars, workshops, and academic conferences attendance. Teachers should be given the opportunity to join professional learning communities through which they can communicate with their colleagues who have experience in gifted education.

### Methodological integrity

As for maintaining the methodological integrity of this study, the data collection included adequate data through involving seven public schools to improve research fidelity and including diverse general and special education teachers in relation to the study goals. Also, the study findings were contextualized within their appropriate content including location and culture. The data led to insights pertinent to the study goals and insightful analyses that promote the utility of the research. As for the data analysis, the findings contribute significantly toward the study goals. Also, the findings are based on data that support understanding, which increases research fidelity.

### Limitations

This study explores special education teachers about gifted preschool education in Abu Dhabi. Thus, collecting similar data from teachers in other UAE emirates might yield different perspectives. Another limitation is the limited teachers’ experience in the field of gifted education in preschool settings.

### Implications for future research

The implications of this study include the importance of providing teachers with several opportunities for professional development that can cater for regular and gifted students’ learning differentiated needs in the classroom. There is a need to provide teachers with assessment tools with which they can screen and nominate students with exceptional abilities in classrooms. Moreover, enrichment programs can be developed in collaboration between teachers and specialists in the field (e.g., university professors and practitioners) to promote preschoolers’ different skills. More research studies are needed to promote the identification and enrichment endeavors for preschool students with exceptional abilities and how gifted preschoolers can transition smoothly from preschool to regular schools.

## Data availability statement

The raw data supporting the conclusions of this article will be made available by the authors, without undue reservation.

## Ethics statement

The studies involving human participants were reviewed and approved by the Social Sciences Ethics Committee of United Arab Emirates University (UAEU). The patients/participants provided their written informed consent to participate in this study.

## Author contributions

Both authors listed have made a substantial, direct, and intellectual contribution to the work, and approved it for publication.
